# The application and deployment of welfare technology in Swedish municipal care: a qualitative study of procurement practices among municipal actors

**DOI:** 10.1186/s12913-021-06944-w

**Published:** 2021-09-06

**Authors:** Sanna Kuoppamäki

**Affiliations:** Department of Biomedical Engineering and Health Systems, KTH Royal Institute of Technology, Stockholm, Sweden

**Keywords:** Welfare technology, Implementation, Procurement, Municipalities, Competence

## Abstract

**Background:**

Welfare technology has been launched as a concept to accelerate digital transformation in care services, but the deployment of these technologies is still hindered by organisational resistance, lack of infrastructure, and juridical and ethical issues. This paper investigates decision-making among municipal actors in the application and deployment of welfare technology from a procurement process perspective. The study explores the perceptions and negotiations involved in purchasing welfare technology at each stage of the procurement model, revealing the impact of technical, economic, juridical and ethical competence on the mapping, planning, procurement, implementation and management of welfare technology.

**Methods:**

The study presents empirical findings from qualitative interviews conducted among municipal actors in Sweden. Semi-structured interviews were gathered in 2020 among procurement managers, IT managers, and managers in social administration in three different municipalities (*n* = 8). Content analysis and systematic categorisation were applied resulting in the division of procurement practices into sub-categories, generic categories and main categories.

**Results:**

Challenges in the application and deployment of welfare technology occur at all stages of the procurement model. In mapping and planning, barriers are identified in the need analysis, requirement specification and market analysis. In the procurement stage, economic resources, standardisation and interoperability hinder the procurement process. Implementation and management are complicated by supplier assessment, legislation, cross-organisational collaboration and political strategy. Building on these findings, this study defines ‘procurement competence’ as consisting of technical, economic, juridical and ethical expertise in order to assess and evaluate welfare technology. Technical and ethical competence is needed in early stages of procurement, whereas juridical and economic competence relates to later stages of the model.

**Conclusions:**

Procurement competence is associated with the application and deployment of welfare technology in (1) assessment of the end-user’s needs, (2) estimation of the costs and benefits of welfare technology and (3) management of juridical and legislative issues in data management. Economic and juridical decisions to purchase welfare technology are not value-neutral, but rather associated with socially shared understandings of technological possibilities in care provision. Optimisation of procurement processes requires a combination of capabilities to introduce, apply and deploy welfare technology that meets the demands and needs of end-users.

## Introduction

Digital transformation and the uptake of welfare technology in care services is expected to improve the quality and cost-effectiveness of care provision. However, the adoption and use of these technologies in municipal care is still hindered by many factors, such as a lack of infrastructure, insufficient resources and juridical and ethical issues [1–4]. Welfare technologies provide assistance in practical tasks, health monitoring, remote treatment and rehabilitation for older adults or persons at risk of disability. They are initiated to optimise the provision of care work, support care delivery and the performance of care-related tasks [2, 5, 6]. These health systems, consisting of safety alarms, night monitoring, digital surveillance, sensor and movement detectors, assistive robots and exercise apps, aim to increase safety, autonomy and independence among older adults. This is expected to result in greater patient empowerment and improved professional practices in health and social care [7].

Research has acknowledged several reasons for the low uptake of welfare technology in municipal care. Welfare technology encompasses a heterogeneous group of technologies that have introduced technologies into novel environments, such as home care services in private homes. The main users of these technologies are older adults, informal and formal care providers [8], and patients with dementia or intellectual disabilities [4]. The deployment of these technologies raises ethical debates on surveillance, quality of life and the assessment of the benefits of welfare technology among different stakeholders [1, 8, 9]. The introduction and application of welfare technology entails adjustments at the individual and organisational levels, which often results in resistance [4, 10, 11]. Adopting and using welfare technologies in care practice implies new ways of working, and changes in professional roles and identities, which require new skills and competencies among health care professionals [12]. Participatory design methods have been applied to aid the implementation of ﻿welfare technology in care settings [13, 14].

Municipalities responsible for purchasing welfare technology in care services often follow a standardised structure of public procurement in Sweden [3, 15]. The procurement, defined as a process by which authorities and other public actors purchase goods, services and public works, can significantly delay or complicate the introduction, application and deployment of welfare technology. Procurement of welfare technology involves different actors, procurement administrators, health care professional and suppliers in the complex task of requirement specification, where technological identities of medical devices are socially constructed and negotiated between different actors [16]. Diffusion and innovation of new medical devices is governed by socio-technical processes and negotiated between healthcare professionals and administrators [17–19]. Purchasing welfare technology also requires managerial and technical skills that range from communication to problem-solving, teamwork, cost analysis and persuasion [20].

Against this background, this paper investigates decision-making complexities among municipal actors in the application and deployment of welfare technology in Swedish municipal care. Based on qualitative interviews in three municipalities, the study analyses how municipal actors negotiate and interpret barriers and enablers of the introduction, application and deployment of welfare technology in relation to the procurement process. Building on these findings, the study conceptualises different layers of knowledge and expertise required during the procurement of welfare technology in municipal care. In conclusion, the study discusses how purchasing welfare technology entails socio-technical understandings of need assessments, cost–benefit analyses and juridical and legislative issues that are interpreted and negotiated in a specific cultural context.

The paper begins by introducing the concepts of welfare technology and procurement in municipal care. This is followed by a presentation of a qualitative analysis of procurement practices, based on semi-structured interviews for municipal actors in Sweden. The results section presents the complexities involved in decision-making in each stage of the procurement model, which results in a definition of ‘procurement competence’ as the economic, technical, juridical and ethical skills needed to assess, evaluate and conduct the procurement of welfare technology.

## Background

### Welfare technology in municipal care

Welfare technology has been initiated as a means of describing the digital transformation of health system changes in Nordic countries [1–3, 5, 9, 15, 21, 22]. Welfare technologies are assistive technologies that provide physical, social and cognitive assistance for older adults and persons at risk of disability, designed to increase their safety, participation and independence, and to improve care delivery and the work environment of healthcare professionals [3, 23]. These technologies include various forms of digital devices, including care robots [24, 25], telecare and alarms [19, 26], monitoring systems [27, 28, 29], digital reminders [30], and mobile health applications [25, 31]. Welfare technologies embody devices such surveillance cameras, key-free locks, GPS alarms, virtual doctors, security bracelets and mobile access to patient documentation and digital signatures for obtaining medication [3], which aim to offer care at a distance, and result in new divisions of labour and responsibility [7].

The introduction, application and deployment of new technical solutions to care practice is not a linear process, and many factors hinder the adoption, use and implementation of welfare technology in municipal care [32]. The deployment of new technological innovations entails changes in healthcare professionals’ work tasks, roles, and identities, which requires management and technological leadership in their organisations. Adopting and using care robots, for instance, influences the work atmosphere, the meaningfulness of work, and the professional development of care professionals, including their orientation, problems with time management and overall attitudes toward renewing service [25]. Confrontations that older adults and their care providers need to negotiate may include technical incompatibilities, professional identities and roles, lack of orientation and fears [33]. For this reason, the deployment of novel technologies is increasingly co-produced, and their application and deployment are often conducted in collaboration between developers and customers. The implementation of new technologies results in different types of organisational, cultural, technological and ethical resistance. This may include, for instance, resistance to chance-established routines, resistance due to language differences, clash of professional cultures, resistance due to patient safety, concerns regarding care quality, patient privacy, dignity, and justice [4].

In Nordic countries, municipalities carry the responsibility to apply, manage, procure and deploy welfare technologies [5, 15]. The application and deployment of welfare technology in municipal care is influenced by a variety of structural factors including procurement legislation, political directives, available resources and competence [2]. According to Socialstyrelsen [34], the majority (78 %) of Swedish municipalities had deployed safety alarms for older people living at home in 2018; the number had risen to 86 % in 2019. Other welfare technologies, such as medication reminders, were implemented by 35 % of Swedish municipalities in 2019, a rising trend from previous years. GPS alarms and night monitoring systems have remained less frequently implemented. In practice, municipalities show great variance in the application and deployment of welfare technology: some municipalities are innovative, testing state-of-the-art innovations, while other municipalities have implemented only the most common welfare technology such as safety alarms [34]. Even though the utilisation of welfare technology is increasing, most welfare technologies have still remained in the test and pilot service stages [35].

### Procurement of welfare technology

Public procurement has been recognised as an important instrument in innovation policy [18, 36, 37]. Procurement is a process whereby authorities and other public actors purchase goods, services, and public works for the purpose of innovating and diffusing new technologies. This has the potential to push innovation forward based on the supply and demand of new technologies [31].

The procurement process is defined in the Swedish Public Procurement Act to ensure that public procurement is based on equality, non-discrimination, openness, mutual recognition and proportionality [38]. Legislation ensures that public procurement is opened up to competition, and that public funds are used effectively. The basic principles of public procurement are objectivity and transparency. Authorities are not permitted to prefer a certain supplier, and the selection of the supplier must be based on open and equal competition. All suppliers must be given the opportunity to compete on equal terms, and supplier selection should be based on which supplier offers the best product in best terms [38]. Additionally, a number of policy documents and recommendations guide the procurement process at the European level [39]. Although the public procurement is a strictly regulated practice, the application of legislation, recommendations and guidelines is expected to vary between municipalities [5].

Procurement is a stage that often delays and hinders the deployment of welfare technology [2, 15, 21]. Nordic countries have developed guidelines and recommendations for the procurement of welfare technology [15, 40]. One of these guidelines is the procurement model created by the National Agency for Public Procurement. The model consists of three chronological steps. The preparation phase (1) includes planning, mapping and analysing the need and the markets for which and from which the technology is to be procured. Municipal actors need to identify and recognise the need for the specific welfare technology, and map out relevant suppliers in the field. This is followed by the procurement phase (2). Municipalities choose either direct procurement or public procurement, based on the value of the procured welfare technology, which will result in an agreement between the supplier and the municipality. The realisation phase (3) involves implementing and managing the procurement of welfare technology by reviewing the delivery.

Procurement processes involve many actors from different organisational levels, which makes it a resource-intensive practice that complicates the application and deployment of welfare technology [3]. Barriers and challenges that prevent a well-functioning procurement process come from organisational structures, mechanisms and processes [16, 41]. An important factor among these is the interaction between procurers and suppliers. Suppliers often lack feedback from procurement managers on previous bids, and therefore have limited possibilities to improve their capacity. Procurement managers show low interest in suppliers’ experience as deliverers to the private sector, and suppliers often have difficulties initiating dialogues with the procurer regarding procedures and conditions [18].

Purchasing welfare technology requires managerial and technical skills, includingas communication, cost analysis, teamwork, problem-solving, negotiation and persuasion [20]. Procurement managers often have low competence levels and limited expertise in conducting complex purchases [42]. This lack of competence hinders the diffusion of innovation [18, 36, 42–44]. The lack of skills and expertise can result in insufficient communication between potential suppliers and procurement actors. Procurement processes involve a variety of actors, who are influenced by social processes, including negotiations regarding the value and purpose of welfare technology [5, 16, 17].

Current research on the deployment of welfare technology in Swedish municipal care has focused on the concept of welfare technology [3], the employment of welfare technology in municipalities [2, 5], digital transformation and welfare technology [22], the views of care personnel on digital transformation [21], and the policies of providing welfare technology [15]. These studies have acknowledged the procurement as one stage that hinders the deployment of welfare technology, but they have not covered the full cycle of the procurement process in relation to the deployment of welfare technology in detail. Furthermore, studies on procurement have shown a connection between procurement results and competence among procurement actors, but these studies have not explored the characteristics of procurement of welfare technology in detail [16, 18–20, 36, 41]. Based on these research gaps, this study investigates the application and deployment of welfare technology from the perspective of the procurement process by focusing on decision-making complexities among municipal actors. These findings will result in a definition of the different dimensions of procurement competence among municipal actors.

## Methods

### Data and participants

The study reports findings from qualitative interviews conducted among municipal actors in three municipalities in Sweden. Data consists of qualitative interviews for eight (8) municipal actors, including procurement managers, IT development managers and managers in social administration (Table 1). The municipalities were selected based on their recent experience in procuring welfare technology. All participants had recently been involved in the application, deployment and procurement of welfare technology. Participants were recruited by sending an e-mail invitation to municipal actors. Interviews were conducted as face-to-face interviews or as phone interviews during the spring of 2020 [35].

**Table 1:** Data characteristics

Municipality	Participant (*n* = 8)	Type of interview
Municipality 1	Procurement manager (2)	Pair interview (face-to-face)
	IT manager (1)	In-person interview (face-to-face)
Municipality 2	Project manager (2)	Pair interview (face-to-face)
Municipality 3	Manager in social administration (2)	In-person interview (phone)
	IT manager (1)	In-person interview (phone)

Data collection was carried out in accordance with guidelines and regulations from Swedish Ethical Review Authority^1^. All participants gave an informed consent for the participation in the study. The study did not collect any personal or sensitive information from participants, nor did it involve any physical intervention, and therefore ethical approval from the Swedish Ethical Review Board was not needed [45]. To protect participants’ privacy, all data has been anonymised, and all identifiers have been removed from the data.

 The interview process followed the principles of qualitative semi-structured interviews. A set of interview questions were designed based on findings from previous literature regarding the application and deployment of welfare technology. The purpose of the interviews was to create an environment where municipal actors felt comfortable sharing their perceptions of the barriers and enablers toof the procurement process. Each interview lasted approximately 60 min. All interviews were recorded and transcribed. The interview questions covered questions regarding municipal actors’ (1) experiences of the procurement process of welfare technology, (2) the decision-making in each stage of the procurement model, (3) perceptions of procurement competence and (4) communication and collaboration with stakeholders. A saturation method was used in each interview: when the interviewers perceived that all relevant information was extracted, the interview session ended [46].

The interview process was informed by the elicitation technique that aims to uncover the tacit knowledge of professionals by providing interviewees concrete reference points so they can recognise, reflect and elaborate upon their perceptions and experiences [47]. As a part of the interview protocol, participants were presented the procurement model of the National Agency for Public Procurement, or they were asked to reflect upon their experiences and decision-making in relation to certain welfare technology, such as night camera or key-free locks. The purpose was to provide interviewees additional information that could facilitate disclosure of understanding and knowledge that would otherwise remain below the surface.

### Data analysis

Transcriptions were analysed with a qualitative content method used for written or visual information with the goal of producing valid inferences from collected data [48]. Content analysis including systematic categorisations was applied as a specific analytical tool to distinguish categories, sub-categories and main categories from collected data. The interpretation of data was performed with regard to the following research questions: (1) how do municipal actors perceive and negotiate the barriers and enablers to the application and deployment of welfare technology with regard to the procurement process, and (2) to what extent can these barriers and enablers be associated with procurement competence in each stage of the procurement model.

In the first phase of the analysis, all transcriptions were read in their entirety. Relevant data extractions were coded with meaning units in each transcript. In the second phase, after the final meaning units were identified, the texts were read once again to check that all data had been accurately coded. In the third phase, meaning units were condensed to sub-categories (*n* = 17), generic categories (*n* = 9) and main categories (*n* = 3). The final phase consisted of summarising the extracted data in a way that was presentable to the reader. Appropriate meaning units were presented as quotations in the text, and a summary of all main categories and sub-categories is presented in Table 2.

**Table 2:** Challenges of procurement practices in different procurement stages

*Procurement stage*	*Generic category*	*Sub-category (data extraction)*
Planning and mapping	Need analysis	Difficult to meet the end-users’ needs Ethical issues regarding privacy Acquisition is not demand-centred Need analysis is not done systematically
	Requirement specification	Difficult to set functional requirements for design Specifying requirements requires skills
	Market analysis	Difficult to select the right product and suppliers Procurement is not governed by end-users’ needs
Procurement	Economic resources	Lack of financial resources
	Standardisation	Lack of national strategy to implement welfare technology Lack of standardisation Lack of integration of welfare technology products Health care responsibilities differ between municipalities
	Interoperability	Requirement for interoperability restricts the amount of suppliers
Implementation and management	Supplier assessment	Difficult to find a suitable supplier
	Cross-organisational collaboration	Need to hire external technical competence
	Political strategy	Lack of political strategy

## Results

This section includes findings on how municipal actors perceive the barriers and challenges of applying and deploying welfare technology in different stages of the procurement model. Planning and mapping consists of decision-making as to the need for the technology, and market analysis and requirement specification. The procurement stage encompasses economic resources, and the need for standardisation and interoperability. Implementation and management refers to supplier assessment, cross-organisational collaboration and political strategies. Building on these findings, this study presents a framework for procurement competence in the application and deployment of welfare technology that consists of technical, economic, juridical and ethical skills and knowledge.

### Planning and mapping

#### Need analysis

The procurement process is regulated by procurement legislation, but each municipal actor performs procurement decisions based on individual considerations. This individual decision-making is performed particularly during the early stages of the procurement process. The complexities of decision-making in the *need analysis* were defined as difficulties to meet end-users’ demands and in specifying requirements. Requirements regarding the ethical aspects of technology design are particularly difficult to fulfill, as patients set more demands than municipal actors are able to deliver:

*You make more and more demands as a patient than we are able to technically deliver to them. The procurement does not say so, but it comes more as a demand from the end users, or those we are trying to help.*

Ethical aspects were perceived to be particularly important when welfare technology was procured for dementia patients or patients with cognitive disabilities. Data storage was considered problematic in the case of GPS alarms and camera sensors that gather and collect patients’ personal data. Ethical requirements also sometimes contradicted functional requirements. Even as welfare technology fulfills functional requirements, it can conflict with patient autonomy. In most cases, ethical decisions in procurement are left for procurement managers to solve:

*There are a lot of ethical problems…we have this with consent. It is very difficult to get consent from people with dementia. The night camera. Shall we look at them while they are lying there sleeping? Safety requirements are also raised when changing to new technology.*

Most participants recognised the importance of the need analysis in the procurement process, but they still lacked the routine of performing it systematically. Need analysis was either based on informal inquiries of healthcare professionals or their managers, or inquiring as to their informal opinions on technological solutions in particular settings. In each case, municipal actors had little or no guidelines as to when and how they should analyse the needs of end-users of welfare technology. Usually the need analysis was based on occasional questions as to the importance of technology, the work environment, and what kind of needs have been raised in their current situations. Home care professionals, in particular, were considered a user group that municipal actors had little or no access to:

*Yes we do (the need analysis), but maybe not as systematically as we should….Either I ask them (health care professionals) to raise the issue further with their managers and come back, or I send them which issues I want them to listen to the employees and ask.*

#### Requirement specification

During the need and market analyses, municipal actors conduct a *requirement specification* that is used to define the technical framework and other requirements for the procured product. This demands a good level of knowledge of both the technical and ethical requirements, and in the design and usability of welfare technology. Welfare technology needs to be attractive and easy to use for end-users, but definitions of usability vary among end-users. Requirement specification was considered difficult, and demanded economic, technical, juridical and ethical competence:

*But if we make a requirement, then this supplier will not be able to deliver. There had been two different providers, one has a cloud service and the other could put it on servers locally. If we want it to be installed locally, and put it as a must-have requirement, but that solution may be a million times more expensive. Then we must know that we need to make this a should-have requirement.*

#### Market analysis

The competence to set the requirements is derived from technical knowledge as to the different functions of welfare technology. Technical competence is particularly relevant in the *market analysis* that refers to the knowledge of relevant suppliers. Knowing which supplier can deliver solutions that fulfil technical demands is a central aspect of competence in market analysis. Another aspect is the *cost–benefit analysis*, where the financial costs of welfare technology need to be carefully considered and optimised in relation to existing benefits. Municipal actors need to have the knowledge on technical requirements to be able define how much it is reasonable to pay for different technological functions, and whether or not it is feasible to invest additional financial resources inin specific technical solutions. This evaluation indicates the optimisation of solutions between the market and user needs, so that requirements can be set in an optimal way. Requirement specification thus combines aspects of both technical and economic competence, and these requirements need to be evaluated from the perspective of end-users and relevant suppliers. The market for welfare technologies is considered broad, which sets a high demand on market knowledge for procurement managers: *We know roughly what we want. But I cannot say that I know what the market can offer because we did not actually do it [the market analysis] in this case*.

In some cases, skimping on market analyses may result in procuring welfare technology that is not based on end-user needs. Municipalities may procure welfare technology based on interests and opportunities, even if these technologies do not necessarily fit the end users’ needs. In particular, municipalities that had good financial resources were driven by an interest to explore new technological solutions. As a result, the evaluation of end-user needs is receiving less attention in decision-making:

*Yes, some of us are very aware of that [procurement is governed by technical possibilities rather than real needs]. With the help of technology, we can measure the heart rate of the person lying down and sleeping and see that they really are alive….But do we need it?*

### Procurement stage

#### Economic resources

Many municipal actors stated a lack of economic resources as a main reason for not procuring welfare technology: *There may be great systems but they are too expensive, so it is almost impossible to use them from an economic perspective even if you see that this has good potential*. The question of economic resources was seen as a political question. In Sweden, politicians in each municipality guide decisions on resource allocation, which can significantly influence economic resources available for elderly care. Economic resources influenced financial capabilities to procure certain technology, but also opportunities to develop *procurement competence* of welfare technology:

*It is about competence because when we may have too many handling errors, it can also contribute to why the technology does not work, so it has absolute competence as well. And it’s about financial resources again, because it costs money to develop the skills of staff.*

#### Standardisation

Procurement competence was primarily associated with technical competence and understanding technical requirements and the possibilities of welfare technology. Participants discussed the need to develop a national strategy to standardise and systematise the procurement process. Current national strategies focus on the procurement process from the legislative perspective, but often lack guidelines on the introduction, application and deployment of welfare technology. While welfare technology is expected to improve the working environments of healthcare professionals and increase the independence and autonomy of end-users, the concrete strategy on how to make use of welfare technology in elderly care was perceived as insufficient:

*When the municipalities procure services in elderly care, it feels as if there is no national or strategic thought about how we should go from where we are today to where people talk about wanting to be.*

Therefore, procurement competence involves the skills, knowledge and understanding of how to utilise welfare technology in an optimal way. This requires understanding care practices and demands for person-centred care among health care professionals. Understanding technical requirements for welfare technology is crucial for being able to select the right type of technology, but in the deployment of technology, the understanding of health care practices is needed to utilise the possibilities of welfare technology. For instance, with certain technological functions, welfare technology can be used to monitor the patient, but to optimise the potential of welfare technology, it should be used to improve the overall well-being of the patient in a more individual and patient-centred way:

*Municipalities must learn to work with the information that different products on the market provide, rather than just the function. Instead of taking a person’s weight every second to see when a person dies, you can use that information to study the person’s movement patterns to see if any other medicine is needed.*

From an organisational perspective, many of the challenges in the procurement of welfare technology can be associated with governmental aspects, including the juridical, legislation and political issues of organising and providing elderly care. The municipalities, guided by municipal politicians, obtain different levels of responsibility in organising health and medical care responsibility for home care. Some municipalities are responsible for providing health care services as a part of home care, and this has a direct influence on procurement decisions: *There are products, e.g. medical reminders where studies show that user organisations save SEK 40,000 per older adult in a year. But there is no interest in municipalities that do not have the health care responsibility to procure such welfare technology*.

#### Interoperability

Juridical issues and legislation complicate the process of procurement due to data management issues and General Data Protection Regulation (GDPR) regulations. Difficult situations can occur when public procurement procedures need to be followed even though only one supplier can deliver a solution, which makes the process time-consuming and inefficient. A specific juridical problem was the *requirement for interoperability* that refers to compatibility between old and new technical systems and their ability to interact. The new technical system needs to fit with other technical systems, which can limit suppliers:

*Requirements for interoperability limit the number of suppliers….A limited number of suppliers means that the entire legal part of the procurement, where suppliers must submit bids and the best price must win, becomes an unnecessary and time-consuming process. It is often only one supplier who will be able to deliver the required solution in the end.*

In a ‘niche’ market like welfare technology, bureaucratic procurement legislation makes it a time-consuming to procure already established welfare technology. The procurement process is based on the assumption that municipal actors research the market, specify the needs and requirements for welfare technology, and chooses the best supplier capable of delivering a product based on specific needs. However, in many cases there is only one supplier that can deliver the required technical solution, but the procurement process needs to be followed according to certain legislation. More flexibility is thus needed to meet the needs of municipal elderly care, and avoid unnecessary delay:

*We know that only this supplier will be able to deliver the product we demand….But we still need to do a public procurement to follow the rules, and then this whole procurement procedure becomes more of a bureaucratic chimera, and this becomes a hindrance and a frustration in a way.*

### Implementation and management

#### Supplier assessment

Procurement practices of welfare technology were also influenced by cross-organisational mechanisms, which refer to the collaboration between municipalities and suppliers. The most important collaboration typically occurs between the municipality and the supplier, but the collaboration between different municipalities is also significant in terms of sharing knowledge, best practices and new opportunities for welfare technology. Regarding the collaboration between the municipality and the supplier, municipal actors articulated difficulties in finding suitable suppliers due to the ‘niche’ market of welfare technology. In many cases, municipalities need to choose the most suitable supplier from four or five options, sometimes settling for the ‘least bad’ supplier: *There is no flawless supplier. It’s a lot (about)…to make sure the supplier does what you want them to do. There is no perfect supplier who knows everything, but it is important to try to find the ones that are the least bad*.

Procurement competence involves good skills and knowledge of the market analysis, and communication with relevant suppliers. When municipalities research suppliers, they often have to deal with a diverse number of companies who offer different sets of technical systems and products. Municipal actors perceive it as challenging that start-up companies do not have knowledge of municipal needs, which makes communication with suppliers difficult. Municipalities need to set specific requirements for procured technology, which also limits the amount of suppliers: *We demand a system that is very good to integrate with our business system, and considering that we have it as the basic requirement, it will only be the same supplier who knows our business system*. Once municipal actors have established contact with the supplier, communication with the suppliers works fluently. Establishing contacts with certain suppliers optimises the procurement process.

#### Cross-organisational collaboration

Collaboration between different municipalities in activities such as market analysis can help municipal actors to optimise the procurement process in terms of knowledge sharing of relevant suppliers and technical competence. Municipalities can reach to other municipalities to consult regarding market analyses and requirement specifications, and to strengthen procurement competence within a single municipality. Knowledge of the relevant suppliers and technical requirements is changing constantly and needs to be updated, and effective communication between municipalities can help with knowledge transformation:

*We wanted this consultant to bring in a competence that was good in understanding what requirements could be set. And you can not have such competence in a municipality. It becomes obsolete again, after a few years.*

#### Political strategy

Collaboration between municipalities can nevertheless be hindered by the lack of existing political strategies in cross-organisational collaboration. The standardisation of procurement practices is needed for companies and suppliers to offer certain products for the market: *Standard, so there must be national standards. These companies we work with would be very happy and say ‘Yes but it is this standard that should be run, and this applies for 20 years*. Political strategies to improve standardisation would also help municipal actors to solve some of the juridical and ethical issues regarding data management and GDPR; the storage of personal information gathered by many welfare technologies such as sensors or cameras. Standardisation is derived from the political strategy to implement welfare technology in each municipality. Political decisions in each municipality influence the investment budget, but they also affect the priorities in municipalities to organise care delivery. Municipal actors mentioned the Scandinavian model in Norway and Denmark as a good alternative, where the deployment of welfare technology is more standardised, and each municipality has less political influence on strategies of welfare technology.

### Procurement competence of welfare technology

This section presents four different dimensions of the procurement competence based on challenges and barriers to the procurement process in each stage of the procurement model. The dimensions of procurement competence were formed based on the analysis of procurement challenges with regard to the type of skills, knowledge and capabilities pronounced by municipal actors. The capability approach to procurement process has identified as higher- and lower-level procurement capabilities [49], and purchasing skills [20]. Lower-level functional capabilities include tasks such as distribution, logistics, and marketing conducted at the individual level, while higher-level dynamic capabilities enable the change, renewal and transformation of existing capabilities to create new products and services at the organisational level [44]. Welfare technology, procured at the municipal sector, has its own requirements for competence that are here defined as technical, economic, juridical and ethical competencies.

*Technical* competence refers to the understanding of the technical requirements of welfare technology in the planning and mapping stage of the procurement model. Data analysis has shown difficulties in requirement specification of welfare technology. This technical competence can be defined as a knowledge of the usability, design and functional requirements of welfare technology. *Economic* competence involves knowledge surrounding conducting the market analysis, recognising relevant suppliers and conducting financial decisions. Economic competence is needed particularly in the procurement stage where financial decisions need to be made based on cost–benefit analysis with a limited amount of financial resources available. *Juridical* competence refers to the knowledge on legislation and juridical issues in conducting the procurement of welfare technology in a systematic and standardised way that takes into consideration the requirement for interoperability and data management. *Ethical* competence relates to need analyses such as recognising and understanding the needs of end-users in early stages of the procurement model, and evaluating the benefits of these technologies for end-users.

## Discussion

 This study has investigated the application and deployment of welfare technology in municipal care from the perspective of procurement practices of welfare technology. This study has explored individual decision-making in each stage of the procurement model with a focus on planning and mapping, procurement and implementation, and management stages of the procurement process. This study complements previous studies on the utilisation of welfare technology in Swedish municipal care by focusing on the procurement task of welfare technology, which has received little attention in previous research [1, 3, 4, 7, 12, 22]. Additionally, this study adds to the literature on procurement practices by describing and defining skills and competencies needed in the procurement of welfare technology [16, 18–20, 36, 41]. To conclude these findings, the study presents the following implications on the procurement of welfare technology to highlight the association between procurement competence and its outcomes.

### Assessment of the end-users’ needs

The application and deployment of welfare technology is a specific practice with regard to the users of this technology. The end-users of welfare technology are typically older adults at risk of disability, and therefore welfare technology may be used by older adult and their informal or formal caregivers [2, 3]. In these complex care situations, the evaluation and assessment of the end-users’ needs becomes a difficult task, sometimes involving conflicting interests and needs between older adults and their care providers. In mapping and planning phase of the procurement of welfare technology, the evaluation of end-users’ needs is often based on occasional and informal inquiries, without a clear systematisation of the standards of the end-users’ needs. Therefore, the technical and ethical competence of procurement of welfare technology, that relates to the understanding of both the technical requirements and the ethical implications of welfare technology, can particularly impact how end-users’ needs are evaluated, and whether or not they are sufficiently taken into consideration in the procurement process. Assessment of end-users’ needs can influence the type of welfare technology that is procured with regard to its translation into requirement specifications [36].

### Estimation of the costs and benefits of welfare technology

The application and deployment of welfare technology involves often decision-making centred around limited economic resources [41]. Technical and economic competence is required to evaluate the costs and benefits of welfare technology for a technically- and economically-optimised solution. Based on this research, technical and economic competence can influence the outcome of procurement, particularly in selecting suppliers. Often the options to find a suitable supplier are limited, and the deployment of welfare technology may be affected by supply-side of procurement, and the type of welfare technologies that currently are available on the market [36]. A cost–benefit estimation that includes skills such as cost management, cost control and cost analysis [20] can nevertheless influence the scope and breadth of procurement. Whether or not welfare technology is seen as a long-term investment for municipal care or a short-term pilot test is influenced by a cost–benefit analysis of welfare technology, and the ability to estimate these in a trustworthy manner.

Although the estimation of costs and benefits is based on economic and financial skills and competencies, this estimation is often associated with social and cultural understandings of the benefits of welfare technology and elderly care. This study concedes that the estimation of costs and benefits is not value-neutral but rather tied to cultural understandings and negotiations of the needs of older adults or a persons at risk of disability, and the benefits that that welfare technology can provide [16, 22]. In Sweden, as well as in other Nordic countries, care provision is influenced by a social norm of independency and autonomy [50]. Welfare technology fits well and advances the policy initiative of aging in place: older adults’ abilities to live independently in their homes for longer [23]. At the same time, however, a social norm of autonomy could complicate decision-making involved in allocating more financial resources to municipal elderly care. From an economic perspective, financial investments for elderly care may be difficult to prioritise at the political level, which could reduce the long-term uptake of welfare technology.

### Management of juridical and legislative issues

The procurementp of welfare technology is a juridical process that is also guided by national and international recommendations and guidelines [34, 38, 39]. These recommendations aim to standardise the process of purchasing welfare technology; in practice, however, procurement actions vary between municipalities. Although municipal actors perceive standardisation important, they consider it to be a political strategy rather than a juridical issue. Legislative and juridical issues in welfare technology are mostly associated with data management and storage; hence they relate to thethe application and deployment of welfare technology more broadly. This study implies that evaluation and assessment of welfare technology should include a question of data storage with respect to end-user privacy. Monitoring systems for fall prevention and detection can offer improved control, independence and safety and reduce the costs of home care services [27, 28]. Sensors that collect data from the location and movements of persons can improve the cost-effectiveness of care provision, but the implementation of these technologies should involve personal choice and consent from the user [28]. For these situations a more specific standardisation and management of juridical issues is needed. The independence and autonomy that welfare technologies can provide should not result in decreased access and availability of other forms of care.

## Conclusions

Welfare technology is a political concept in the Nordic countries that is expected to improve quality of care in municipal care [1–3, 5, 9, 15, 21, 22]. This study has taken a specific outlook on the application and deployment of welfare technology by focusing on municipal actors’ perceptions and experiences in the procurement process. With a focus on negotiation, understanding and decision-making during the procurement practices, this study has shown the barriers to purchasing welfare technology with regard to skills and competencies outlined by municipal actors. Building on these findings, this study argues that economic and juridical decisions in purchasing welfare technology are not value-neutral, nor strictly determined by technical skills, but also associated with ethical and sociocultural understandings of the role of technology in care provision, delivery and care-related tasks.

Although the data only covered a limited number of participants and municipalities, the analysis clarified the skills and competencies with regard to purchasing welfare technology. These skills are essential in the application of legislation considering purchasing welfare technology. Future research should take a more comprehensive outlook on the mechanisms and interactions between suppliers and municipal actors and investigate procurement practices from the supplier’s perspective. Application of legislation could be investigated in relation to other regulatory practices, and their connections to diffusion of innovation in municipal care. Furthermore, challenges and barriers outlined in this study should be investigated from a comparative perspective that could give insights as to the scope and frequency of these challenges in the application of legislation between countries.

## Data Availability

The datasets used and analysed during the current study are available from the corresponding author on reasonable request .

## Footnotes

1 An ethical approval is needed only if: 1)the study includes processing of personal or sensitive personal data. This information include racial or ethnic origin, political opinions, religious or philosophical beliefs, trade union membership, health, or sexual orientation; 2) the study collects biological material that has been taken from a natural person and can be traced to this person; 3) the study involves a physical intervention or uses a method that aims to affect the researcher physically or mentally [45].
